# Heritability of the extra-pair mating behaviour of the pied flycatcher in Western Siberia

**DOI:** 10.7717/peerj.9571

**Published:** 2020-07-31

**Authors:** Vladimir G. Grinkov, Andreas Bauer, Helmut Sternberg, Michael Wink

**Affiliations:** 1 Evolutionary Biology Department, Faculty of Biology, Lomonosov Moscow State University, Moscow, Russian Federation; 2 Tomsk State University, Tomsk, Russian Federation; 3 Institute of Pharmacy and Molecular Biotechnology, Heidelberg University, Heidelberg, Germany; 4 OAG f. Populationsforschung Braunschweig, Braunschweig, Germany

**Keywords:** Animal model, Extra-pair copulations, Extra-pair paternity, Heritability, Pied Flycatcher, *Ficedula hypoleuca*

## Abstract

Males and females take part in extra-pair copulations in most socially monogamous bird species. The mechanisms leading to the frequent occurrence of extra-pair offspring in socially monogamous couples are strongly debated and unresolved, and they are often difficult to distinguish from one another. Most hypotheses explaining the evolution of extra-pair reproduction suggest selective and adaptive scenarios for their origination and persistence. Is extra-pair paternity a heritable trait? We evaluated the heritability of extra-pair paternity in the pied flycatcher (*Ficedula hypoleuca*) nesting in Western Siberia. Estimated heritability was low: depending on the model used, the point estimate of the heritability (mode) varied from 0.005 to 0.11, and the bounds of the 95% confidence interval are [0–0.16] in the widest range. Thus, it seems that extra-pair mating behaviour in the pied flycatchers is a plastic phenotypic mating tactic with a small or no genetic component. Our data can help to understand the evolution of extra-pair mating behaviour in socially monogamous species.

## Introduction

DNA profiling (Jeffreys, 1987) has revealed that males and females take part in extra-pair copulations (EPCs) in many socially monogamous bird species (Wink & Dyrcz, 1999; Griffith, Owens & Thuman, 2002; Westneat & Stewart, 2003). In case of successful EPCs, extra-pair offspring (EPO) could be detected in broods of socially monogamous families. The percentage of EPO in a brood, and especially the proportion of those couples whose broods have EPO, can vary widely even within one species. For example in the pied flycatcher (*Ficedula hypoleuca*), the proportion of broods containing EPO varies from 6.5% to 40% between populations (Lifjeld, Slagsvold & Lampe, 1991; Rätti et al., 1995; Lubjuhn et al., 2000; Slagsvold et al., 2001; Lehtonen, Primmer & Laaksonen, 2009; Moreno et al., 2010, 2015; Canal, Jovani & Potti, 2012; De la Hera et al., 2013; González-Braojos et al., 2013; Tomotani et al., 2017; Grinkov et al., 2018).

The mechanisms leading to the frequent occurrence of EPO in socially monogamous couples are strongly debated (Halliday & Arnold, 1987; Sherman & Westneat, 1988; Jennions & Petrie, 2000; Arnqvist & Kirkpatrick, 2005, 2007; Kokko, Jennions & Brooks, 2006; Griffith, 2007; Kempenaers, 2007; Eliassen & Jørgensen, 2014; Forstmeier et al., 2014). Most hypotheses explaining the evolution of extra-pair reproduction suggest selective and adaptive scenarios for the origin and persistence of extra-pair mating behaviour (for review, Eliassen & Jørgensen, 2014; Forstmeier et al., 2014; Lifjeld et al., 2019; Brouwer & Griffith, 2019). Selection acts on phenotypes, and it only has evolutionary consequences when fitness differences among individuals relate directly to genetic differences. Therefore, for selection to effectively control the evolution of extra-pair paternity, a genetic component should be present in the variation of traits associated with extra-pair reproduction (Hill, Goddard & Visscher, 2008).

In general, the change in the mean value of traits between generations under natural selection on short-term scale happens if the traits are heritable and the trait value associates with fitness (Fisher, 1918). Therefore, models describing changes in a trait under natural selection include three components: trait related differences in fitness among individuals, the heritability of the trait and the amount of variation in the trait. The basic phenotypic model of natural selection defines that the change in the mean value, $\Delta \bar z$, of a trait, *z*, is $\Delta \bar z = s \times {\rm g}/{\rm{\sigma}} _z^2$ where $s$ is the selection differential, g is the genetic component (commonly represented as the additive genetic variance in the trait, ${\rm {\sigma}}_A^2$) and ${\rm{\sigma}} _z^2$ is the total phenotypic variation in a trait (Lande, 1979; Lande & Arnold, 1983). The ratio ${\rm g}/{\rm{\sigma}} _z^2$ is defined as narrow-sense heritability, *h*^2^ (if ${\rm g} = {\rm{\sigma}}_A^2$). The change in the mean trait value could be defined as the response to selection, *R*. Therefore, the simple ‘breeder’s equation’ is $R = {h^2} \times s$ (Robertson, 1966; Price, 1970; Falconer & MacKay, 1996). In natural populations, the selection differential can be expressed as the covariance ${\rm Cov}\left( {{\rm{\omega}} ,z} \right)$ between the trait and fitness, ω (Lande & Arnold, 1983). Consequently, the change of trait between generations is $\Delta \bar z = {\rm g} \times {\rm Cov}\left( {{\rm{\omega}} ,z} \right)/{\rm{\sigma}} _z^2$. Because the regression coefficient, *b*, relating fitness to the trait, equals to ${\rm Cov}\left( {{\rm{\omega}} ,z} \right)/{\rm{\sigma}} _z^2$, the change of trait between generations is

(1) $$\Delta \bar z = {h^2} \times {\rm Cov}\left( {{\rm{\omega}} ,z} \right) = {\rm{\sigma}} _A^2 \times b$$

In other words, the per-generation change in the mean of a quantitative trait caused by selection is the product of the selection gradient and the additive genetic variance (Robertson, 1966; Price, 1970; Lande, 1979; Lande & Arnold, 1983; Falconer & MacKay, 1996).

In the case of extra-pair reproduction, these general equations can be converted to estimate the strength of natural selection for an extra-pair mating behaviour for both males and females. It is commonly accepted that the EPO number reflects EPC behaviour of an individual (Arnqvist & Kirkpatrick, 2005, 2007; but see Dunn & Lifjeld, 1994; Griffith, 2007). Therefore, the change in the mean rate of EPC approximated as the change in the mean EPO number, $\Delta {\bar N_{\rm EPO}}$, under natural selection is simply followed from Eq. (1) for both sexes, that is

(2) $$\Delta {\bar N_{\rm EPO}} = h_{{N_{\rm EPO}}}^2 \times {\rm Cov}\left( {{\rm{\omega}} ,{N_{\rm EPO}}} \right)$$

where $h_{{N_{\rm EPO}}}^2$ is the heritability of the EPO number, ${\rm Cov}\left( {{\rm{\omega}} ,{N_{\rm EPO}}} \right)$ is the covariance between the EPO number and individual fitness. In males of socially monogamous species, this equation can be relatively easily understood because extra-pair paternity (EPP) directly increases an individual male’s reproductive success and hence one of the main components of fitness, fecundity (Trivers, 1972). However, a simple linear covariance between the number of EPO and the fitness of the individual is not always possible. For males, for example, an increase in the number of EPO cannot always be associated only with an increase in fitness. At some point, the time allocated to find extra-pair females may lead to both a loss of within-pair paternity and a decrease in parental care for within-pair offspring. The emergence of such a trade-off can be considered by modification of the selection differential: the linear selection differential can be replaced by the quadratic selection differential (for details, please, refer to Henshaw & Zemel (2017)). At the same time, the forces driving extra-pair reproduction by socially monogamous females are less clear because EPC is not associated with an increase of a female’s immediate reproductive success since female’s EPC does not necessarily increase fecundity. This is because females’ reproductive output is limited by their reproductive biology rather than by their number of mates. There are the potential direct fitness benefits for females involving in EPCs (for example, fertilisation assurance of eggs, nuptial gifts from several mates, increased paternal care at nest, cooperative neighbourhood) (Stacey, 1982; Davies, 1992; Sheldon, 1994; Davies et al., 1996; Arnqvist & Nilsson, 2000; Tryjanowski & Hromada, 2005; Eliassen & Jørgensen, 2014) as well as the potential direct costs (sexually transmitted disease, reduced paternal care by the within-pair social mate) (Beemer, Kuttin & Katz, 1973; Davies, 1992; Sheldon, 1993; Davies et al., 1996; Lombardo & Thorpe, 2000; Houston, Szekely & McNamara, 2005). Strong natural selection would seem to effectively eliminate the variation in infertility in individuals (but see, e.g. Morrow, Arnqvist & Pitcher, 2002), and it is therefore sometimes suggested that the direct fitness benefits for females participating in the EPC may often be less obvious than the direct costs (Griffith, 2007). On the other hand, females involving in EPCs in birds can take advantage of indirect genetic benefits (Wink & Dyrcz, 1999). Females could seek copulations with extra-pair males of superior genetic makeup to increase offspring fitness (Jennions & Petrie, 2000), or because genetically complementary males may sire heterozygous offspring with potentially higher fitness (e.g. due to improved immuno-competence) (Kempenaers, 2007). Therefore, extra-pair reproduction in females is hypothesised to be governed by female-specific indirect and direct selection (Kirkpatrick & Barton, 1997; Arnqvist & Kirkpatrick, 2005; Kokko, Jennions & Brooks, 2006).

The equations approximating the force of different types of selection on female extra-pair mating behaviour are generally similar for sex unspecific ones Eq. (2). As noted above, these equations contain two common components: heritability and estimate of the total variability of the trait. They differ in the method of calculating the third component, the selection gradient or the selection differential. Again, assuming that the EPO number reflects female extra-pair mating behaviour, the change in the mean EPO number under the influence of *indirect selection*, measured in units of phenotypic standard deviations, is ${\Delta _I}{\bar N_{\rm EPO}} = h_{{N_{\rm EPO}}}^2 \times {{\rm{\sigma}} _{{N_{\rm EPO}}}} \times {s_{\rm EW}}$, where $h_{{N_{\rm EPO}}}^2$ is the heritability of the EPO number, ${\sigma _{{N_{\rm EPO}}}}$ is the phenotypic standard deviation of the EPO number, and ${s_{\rm EW}}$ is the difference in fitness between EPO and within-pair offspring (WPO) (Kirkpatrick & Barton, 1997; Arnqvist & Kirkpatrick, 2005, 2007; but see Griffith, 2007). The per-generation change in the mean rate of female’s EPC caused by *direct selection*, measured in units of phenotypic standard deviations, is ${\Delta _D}{\bar N_{\rm EPO}} = \textstyle{1 \over 2} \times h_{{N_{\rm EPO}}}^2 \times {{\rm{\sigma}} _{{N_{\rm EPO}}}} \times {{\rm{\beta}}_{{f_{\rm EPC}}}}$ where ${{\rm{\beta}}_{{f_{\rm EPC}}}}$ is the direct selection gradient acting on a female’s propensity to engage in EPC that results from reduced paternal care of her social mate (Arnqvist & Kirkpatrick, 2005, 2007). This formula is correct if one agrees that ‘Reduced parental care is also the only form of direct effect of female EPC behaviour whose impact can currently be quantified’ (Arnqvist & Kirkpatrick, 2005, 2007; but again, see Griffith, 2007).

In our work, we did not aim to assess the balance of forces between direct and indirect selection. We have given Eq. (2), as well as formulas for different types of selection in females approximating the change in the mean rate of their EPC to demonstrate the importance of the narrow-sense heritability for selective mechanisms of trait evolution. All equations have very well demonstrated that selection depends on heritability and additive genetic variance. Perhaps, the biological meaning of these equations is that resemblance between relatives is mostly driven by additive genetic variance (Hill, Goddard & Visscher, 2008).

Nevertheless, despite the importance of key genetic and phenotypic variances and covariances to all hypotheses pertinent to the selective mechanisms driving extra-pair reproduction in socially monogamous species, an explicit estimation of heritability of EPC behaviour has been rarely made. We know only two animal species for which genetic variances of EPP rate have been dissected, namely for the song sparrow (*Melospiza melodia*) (Reid et al., 2011a, 2011b) and humans (*Homo sapiens*) (Zietsch et al., 2015). Using data on extrapair mating in 7,378 Finnish twins and their siblings, it has been shown that the within-sex broad-sense heritability (the percentage of variation in extrapair mating due to total genetic variation, not only additive component) is 62% in men and 40% in women (Zietsch et al., 2015). In *M. melodia*, estimates of ${\rm{\sigma}}_A^2$ and *h*^2^ were both close to 0 in males (Reid et al., 2011b), and were 1.08 and 0.12 for ${\rm{\sigma}}_A^2$ and *h*^2^, respectively, in females (Reid et al., 2011a). Such a small number of data for vertebrates is not particularly satisfactory given the important theoretical and practical significance of heritability in our understanding of micro- and meso-evolution, formalised in Eq. (1). Knowledge of the key genetic components of variation in the extra-pair reproduction can enact a discriminating criterion when choosing hypotheses about the evolution of extra-pair mating behaviour for each specific study of birds. Indeed, the number of hypotheses about the evolution of EPC incidence is huge, and the proposed mechanisms are often difficult to distinguish from one another (Ford, 1983).

Therefore, here we evaluate the additive genetic variance in the extra-pair reproduction of the pied flycatcher (*Ficedula hypoleuca*) breeding in Western Siberia and its proportion in the total phenotypic variance of the trait, narrow-sense heritability, *h*^2^. Hence, estimating *h*^2^, we can qualitatively evaluate the magnitude of the selective forces that are necessary to drive the evolution of extra-pair reproduction in the pied flycatcher. Thus, we test the hypothesis of whether the population retained the extra-pair mating behaviour due to natural selection acting indirectly on the EPC through an associated trait such as the EPO number.

## Materials and Methods

### General approach

Usually, to assess the heritability in natural populations, multi-generation longitudinal studies are carried out to accumulate enough empirical phenotypic data to estimate a resemblance between relatives in target traits. However, if the recruitment rate of marked young is high in a local population, then in each breeding season, the population will include both parents and their descendants, which have survived until the year of study from previous reproductive seasons. Therefore, if we carry out single-year cross-generation measurements of a target trait in all specimens in the studied population, then we will be able to identify relatives and compare their values of the investigated trait. We already have successfully applied this approach to assess the heritability of the basal metabolic rate in the pied flycatcher (Bushuev et al., 2012), because in the Western Siberian population of the pied flycatcher the recruitment rate of locally born individuals is on average 11.1% both in males and females (Grinkov & Sternberg, 2018).

For natural populations, phenotypic measures are available for individuals with a mixture of genetic relationships (number of known relatives) within and across multiple generations (the unbalanced design). Therefore, an additive genetic variance and environmental components could be estimated most efficiently from a linear mixed model, the ‘animal model’ (Henderson, 1953; Kruuk, 2004; De Villemereuil, Gimenez & Doligez, 2013). In this model, all pairwise additive genetic relationships in the entire pedigree are used and, for analysis, all sources of information are appropriately weighted by their sampling variance (Visscher, Hill & Wray, 2008).

### Study species and population

Our research was conducted on the pied flycatcher. The pied flycatcher is one of the more common ‘Old World’ forest-dwelling bird species. The breeding area of the species extends from Spanish Malaga and Moralech in the west to Russian Krasnoyarsk in the east. The breeding range of the species is restricted to the forest zone in the south and the north. The pied flycatchers migrate to sub-equatorial western Africa for wintering. Commonly, the pied flycatchers nest once a year and make a second breeding attempt in the case of an unsuccessful first nesting. Flycatchers form a pair bond for the current breeding season and change social partners with each subsequent reproduction event (serial monogamy). Some individuals have polygamous relationships within one breeding season (Grinkov et al., 2018).

The individual-level study of the pied flycatcher population breeding in the Tomsk region, Western Siberia, Russia (56°21N, 84°56E) has been running with constant research effort and identical working methods since 2001. Immigrant flycatchers to the study population were captured, ringed and released; they can be identified individually throughout a lifetime. Flycatchers, born inside the population, were ringed and monitored from birth through all breeding attempts until they disappeared (when they probably died).

The studied pied flycatchers breed in nest-boxes. The nest-box area is located 13 km south of Tomsk in a mixed small-leaf deciduous forest, which mainly consists of aspen (*Populus tremula*) and birches (*Betula* spp.) which is typical for the southern taiga subzone. The pied flycatchers build nests in natural holes, but strongly prefer artificial nest-boxes over natural cavities. Nest-boxes facilitate the trapping of almost all adult breeders of the studied breeding population. The nest-box area consists of three plots—two 5 ha and one 20 ha areas. The distance between the most distant nest-boxes in the study area is approximately 3 km. Over the years, the number of monitored nest-boxes varied depending on short-term experiments. We always had more than 200 and less than 381 nest-boxes under observation. The number of breeding flycatchers is strongly dependent on the number of nest-boxes. In Western Siberia, the pied flycatcher has no competitors among other bird species which would compete with the nest-boxes. The occupation rate of nest-boxes could reach 96%, thus the size of the breeding population, though fluctuating from year to year, was not less than 100 pairs (the maximum was more than 320 pairs). The monitoring scheme of the studied population was based on regular nest-box inspections to record breeding time, estimate fecundity, and determine catching dates of adult birds. We captured and ringed almost all females two times during the breeding season: for the first time each female was caught between day 7 and 9 of clutch incubation, and for the second time a female was caught feeding 9‒11-day-old nestlings. Males were caught feeding their nestlings at the age of 9‒11 days. More detailed information about the monitoring scheme of the population, the frequency of bird catching, their treatment, as well as a detailed description of the research area can be found in our earlier publication (Grinkov et al., 2018).

### Measurement of the EPO number

In 2005, using brachial (wing) venipuncture we collected blood samples from all birds breeding in the study area for paternity analyses. Males and females were blood-sampled when feeding 9–11-day-old nestlings, nestlings were blood sampled at day 10 to 12 after hatching (the next day after blood sampling from parents). We sampled 232 males, 250 females, 1,485 nestlings (250 nests; 1,967 blood samples). The collected blood samples were stored in an EDTA-buffer. After DNA extraction the pied flycatcher samples were genotyped using eight microsatellite loci FHU1/PTC2, FHU2/PTC3 (Ellegren, 1992); FHU3, FHU5 (Primmer, Møller & Ellegren, 1996); FHY336, FHY403, FHY427, FHY452 (Leder, Karaiskou & Primmer, 2008). The microsatellite loci were amplified using two multiplexes PCRs (Set FHU, Set FHY). Amplified fragments were analysed via capillary electrophoresis (MegaBACE 500 Sequencing System and MegaBACE 1000 Sequencing System; GE Healthcare Europe GmbH). The degree of genetic relationship was determined by CERVUS 3.0 (Kalinowski, Taper & Marshall, 2007). All work for paternity analysis was conducted at the Institute of Pharmacy and Molecular Biotechnology at Heidelberg University in Germany. For characteristics of the molecular markers used in the study and for a detailed description of the assessment of the paternity of each nestling, we refer to our previous publication (Grinkov et al., 2018).

For the present study, we assume that the number of offspring sired by an extra-pair male is a good phenotypic measure of the extra-pair mating behaviour. This means that we evaluate the selective forces acting on the EPP rate rather than on the EPC rate because a direct registration of copulation behaviour of all birds breeding in the study area was impossible in our case. In general, a linear dependence between the observed rate of EPCs and EPP was not demonstrated across an analysed species (Dunn & Lifjeld, 1994; Griffith, 2007). However, it seems that in the case of the pied flycatcher there is a covariance between the observed rate of the EPP and the true rate of EPCs. In the studied population, females can have up to three extra-pair sires (Grinkov et al., 2018). At least among 53 females with EPO, the EPO number and the EPP rate (this is the ratio of EPO to the number of nestlings in the brood) positively correlated with the number of extra-pair males (Pearson’s product-moment correlation, *r* = 0.40, *p* < 0.003, and *r* = 0.40, *p* < 0.003, respectively). It is most likely that the females having EPO from three extra-pair sires had more EPCs (minimum three EPCs) than the females having EPO from one extra-pair sire (minimum one EPC). This is also true for 40 males with EPO, there is a linear correlation between the EPO number and the extra-pair female number (*r* = 0.60, *p* < 0.0001). Nonetheless, we are unable to assume that females and males without EPP have not had EPCs at all. However, based on these correlations, the assumption that EPP correlates positively with the degree of EPC behaviour across females and males within populations looks reliable and parsimonious (Arnqvist & Kirkpatrick, 2007).

### Fixed and random effects

In the linear mixed models, we included the most important and most common fixed and random effects, which are usually modelled when assessing the heritability value. We conducted a preliminary exploratory analysis (Grinkov et al., 2014) and found that age for males and breeding site for females were associated with the EPO number, and females and males did not differ in the average EPO number. Two factors were a binary variable, that is all birds ‘males’ and ‘females’ were ‘yearlings’ or ‘older’. The quality of the breeding site was a numeric variable containing only integer values, that is number of recruits. Sex was determined by morphological traits using colouration patterns of breeding plumage and an incubation patch developed only by females of the species. Age was determined according to the ringing data, and the territory quality was determined by the recruitment number that grew in a given nest-box for the period spanning from 2001 to 2009 (±4 years to the year of the EPO number measurement). Age and sex entered in models as fixed effects and the quality of the breeding site as a random effect.

Heritability estimates are known to be critically determined by the fixed effects structure of the model used (Wilson, 2008): each added fixed effect could possibly result in an artificial raise of heritability. It is believed that if ${\rm{\sigma}}_A^2$ is scaled by the observed phenotypic variance in the data, then heritability appears to be relatively constant across models (Wilson, 2008). Apparently, this is not always the case, however, scaling can at least partly alleviate the issues associated with fixed effects structure in models. Therefore, we did not pay much attention to the statistical testing of the significance of the fixed effects and did not use conventional model selection procedures based on the deviance information criterion (DIC) associated to the model (Wilson, 2008). Alternatively, as recommended (Wilson, 2008) we constructed a series of animal models differing in their fixed effects structure (including models without fixed effects) to calculate the additive genetic variance. This allows tracing changes in ${\rm{\sigma}}_A^2$ caused by fixed effects structure, and it helps to make a choice between models and to obtain an unbiased assessment of heritability as close as possible to the actual heritability in the population. Later, if it was necessary to estimate the strength of selection, it should be estimated in a way that *h*^2^ was conditioned on fixed effects (Wilson, 2008).

Also, here it should be noted that the structure of the animal model may produce a downward biased estimate of the average additive genetic variance. For example, we can construct the univariate model including sexes as a fixed effect. A univariate analysis of this type incorporates the assumptions of no genotype-by-sex interactions and, therefore, a between-sex additive genetic correlation of one (Wolak, Roff & Fairbairn, 2015). When these assumptions are valid mixed effect models treating any differences between the sexes as a fixed will produce unbiased estimates of the additive genetic variance in the population (Wolak, Roff & Fairbairn, 2015). However, if the between-sexes additive genetic correlation is less than unity, the univariate model will produce a biased estimate of additive genetic variance: the average variance will be less than either of the two variances (for males or females) (Wolak, Roff & Fairbairn, 2015). Therefore, we also conducted additional calculations to estimate the heritability for both sexes separately to check whether there was a downward bias in the joint estimates of the average additive genetic variances in the univariate models including sex as a fixed effect.

### Pedigree construction and statistical analyses

We need knowledge about the genetic relatedness among individuals to conduct a quantitative genetic analysis. It is common in avian studies that kinship is inferred based on catching the females when they incubate eggs and the males when they provide food for their young in the nests (Grinkov et al., 2018). There may be some errors through the paternal line because of alloparental care, which is any form of parental care directed towards non-descendant young (Wilson, 1975; Grinkov et al., 2018). EPCs are the main source of alloparents in a population, and they potentially may cause that paternal links within the social pedigree may differ from those in the actual genetic pedigree, and therefore using social pedigrees for quantitative genetic parameter estimation may be incorrect. The simulation studies showed that the random EPP leads to an underestimation of the heritability, but the extent of underestimation remained small (Charmantier & Reale, 2005). It was concluded that social pedigree was generally reliable for quantitative genetic analysis when EPP rates ≤20% (Charmantier & Reale, 2005; Firth et al., 2015). In case of the non-random EPP, low heritability traits appear to be relatively unaffected by EPP, and, even for traits with higher heritability, the social pedigree remains adequate in most cases (Firth et al., 2015). Regarding other sources of alloparents in the pied flycatcher, it seems that similar conclusions can be made since their occurrence is even lower in the population (Grinkov et al., 2018).

We used the open-source R software environment for statistical computing and graphics (version 3.5.0) under an integrated development environment for R—RStudio (RStudio Desktop version 1.1.447) to analyse data. We used the R package ‘pedantics’ to search for errors in the pedigree, correct them and obtain summary statistics (Morrissey & Wilson, 2009). The initial pedigree consisted of 2,878 individuals (930 maternities, 905 paternities). There were 7 ancestral generations for individuals (mean 0.56, minimum 0, maximum 6). The distribution of known relatedness among individuals is (relatedness value–number (only non-zero values)) 0–4133145, 0.025–499, 0.05–808, 0.075–2, 0.125–1,435, 0.15–2, 0.175–1, 0.25–1979, 0.3–1, 0.375–5, 0.5–2124, 0.625–2. The number of informative individuals, ‘pruned pedigree’, for the quantitative genetic analyses of the EPO number consisted in total of 577 individuals spanning four generations (mean 0.41, minimum 0, maximum 3) where 134 were maternities, 132 were paternities (33 full sibs; 69 maternal sibs; 36 maternal half-sibs; 66 paternal sibs; 33 paternal half-sibs). The maternal (paternal) sibs number is the total number of pair-wise maternal (paternal) sib relationships defined by the pedigree; to get the number of maternal (paternal) sibs sum number of full sibs and maternal (paternal) half-sibs, respectively (please, refer to ‘pedantics’ package manual). These numbers of different types of relatedness correspond to many small families because most couples produce (if they do) only one or two recruits. For example, 134 maternities include 78 mother-to-child relationships, where 34 mothers (43.6%) have one recruited offspring, 33 mothers (42.3%) have 2 offspring, 10 mothers (12.8%) have 3 offspring, and finally one mother (1.3%) has 4 offspring. Similarly, 132 paternities include 76 father-to-child relationships in which 30 fathers (39.5%) have one recruited offspring, 36 (47.4%) fathers have 2 offspring, and 10 fathers (13.1%) have 3 offspring. Therefore, mean maternal sibship size was 1.72, mean paternal sibship size 1.74. The distribution of known relatedness among individuals of pruned pedigree is (relatedness value–number (only non-zero values)) 0–165555, 0.025–6, 0.05–36, 0.125–97, 0.25–183, 0.5–299; and mean pairwise relatedness of all individuals in the pruned pedigree was 0.0013.

We used the Markov chain Monte Carlo method for estimating genetic variance components as it was implemented in MCMCglmm package (Hadfield, 2010) in R, because it is flexible, and allows Bayesian estimation in complex pedigrees.

**Figure 1:**
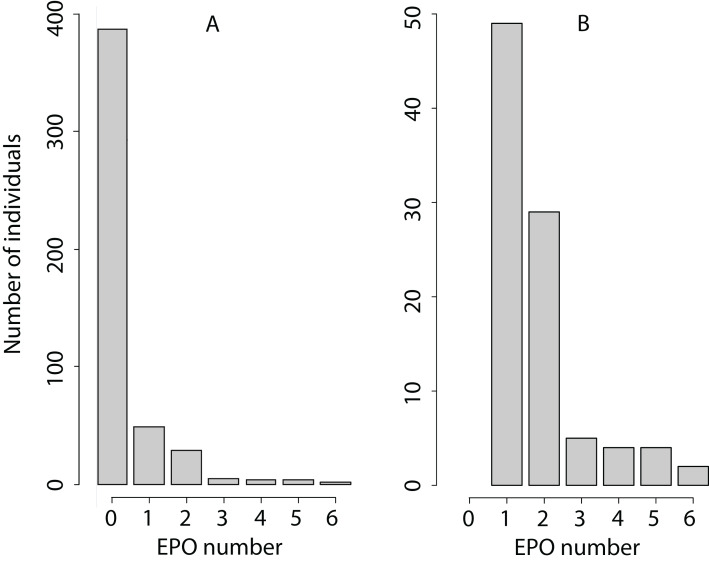
Phenotypic variation in the EPO number. (A) The number of individuals with each EPO value is shown. (B) Only the number of individuals with the EPO value > 0 is shown to increase the resolution of the graph for EPO values exceeding 0.

The EPO number is a non-negative integer with a very high proportion of zeroes (Fig. 1), with mean 0.35, variance 0.81, and maximum value 6 EPO (sample size 480: 250 females and 230 males). Therefore, we analysed the EPO number assuming an overdispersed Poisson distribution (as an example of the analysis of such traits, see Kruuk, Clutton-Brock & Pemberton, 2014). MCMCglmm fits an additive overdispersion model. It uses a log-link and estimates variance components on the latent scale. We conducted posterior predictive checks as is described in MCMCglmm course notes, Section 5.3.1 (Hadfield, 2018). We found that this overdispersed Poisson distribution was sufficient to represent the distribution of the EPO number because the observed and mean predicted number of zeroes differed by 2–4%.

The choice of a prior distribution for a Bayesian model is a difficult subject (De Villemereuil, Gimenez & Doligez, 2013; Morrissey et al., 2013; Hadfield, 2018). Therefore, in our work, we used the prior distribution taken from other similar studies (Reid et al., 2011a). We used default MCMCglmm priors for fixed effects (normally distributed, diffuse, with mean 0 and variance 1 × 10^8^). Parameter-expanded random effects priors were G1 (V = 1, nu = 1, alpha.mu = 0, alpha.V = 1,000), and G2 (V = diag(2), nu = 1, alpha.mu = rep (0,2), alpha.V = diag (2)*1,000). The prior for the residual covariance structure was R1 (V = 1, nu = 1) and with fixed variance structure R2 (V = 1, fix=1). All models were run with 1,005 × 10^4^ number of MCMC iterations with a thinning interval of 10^4^ and a burn-in period of 5 × 10^4^, resulting in a total sample size of 1,000 estimates. The effective sample sizes for each estimate in all models were 1,000. The convergence of the MCMC sampling was assessed by visual inspection of the variance component chains; autocorrelation between successive observations was <0.12 across all analyses.

We calculated heritabilities on the latent scale as ${\rm{\sigma}}_A^2/\left( {{\rm{\sigma}}_A^2 + {\rm{\sigma}}_R^2} \right)$ where the residual variance, ${\rm{\sigma}} _R^2$, is the sum of all variance components depending on the model structure except ${\rm{\sigma}}_A^2$ (Morrissey et al., 2013). However, it should be remembered that in all models where fixed effects have been included (in our case it is sex and age) the sum ${\rm{\sigma}}_A^2 + {\rm{\sigma}}_R^2$ does not constitute the entire observed phenotypic variance: the sum of random variances (additive genetic, random effects and residual variances) reflects the variance after the fixed effects have been accounted for Wilson (2008), De Villemereuil (2012), and De Villemereuil et al. (2018).

Because the additive variance is not the same on the latent and data scales, there is no easy way to calculate heritability on the data scale (Morrissey et al., 2013). Therefore, we calculated heritability on data scale using the QGglmm package in R (De Villemereuil et al., 2016; De Villemereuil, 2018). It allows for the computation of the quantitative genetics parameters on the observed data scale after a Generalised Linear Mixed Model (GLMM) analysis (De Villemereuil et al., 2016). The QGglmm R package is especially useful in practice, because it also allows accounting for the ‘fixed-effect’ variance component in computation the quantitative genetic parameters on the observed data scale in models in which the studied traits are not normally distributed.

We simulated the zero-heritability trait following Poisson distribution using the pedantics package. All models were also fitted with this ‘null’ trait, and the resulting heritabilities were calculated with the above-described procedure. The obtained estimates of heritability for the observed trait were compared with the estimates of the ‘null’ trait, to conclude that the heritability of the observed trait is not equal to zero, because in MCMCglmm all variance components cannot overlap zero.

We used the highest posterior density (HPD) region (Hyndman, 1996) for 95% limits as a descriptor of 95% credible interval (CI) to summarise variation of *h*^2^. HPDs were calculated using the LaplacesDemon package (Statisticat, LLC, 2020) in R. In case of the unimodal bell-shaped distribution, HPD region coincides with a 95% quantile-based probability interval but in case of more complex polymodal distributions they could significantly differ.

### Ethics statement

Our work conforms to the legal requirements of the Russian Federation as well as to international ethical standards. All our treatments and samplings have been intravital and have not required prolonged treatment and handling of birds. The species from our study is not included in the ‘Threatened’ category of the IUCN Red List of Threatened Species. The Bioethics Commission of Lomonosov Moscow State University has provided full approval for this research (Protocol No 89-o of March 22, 2018).

## Results

**Figure 2:**
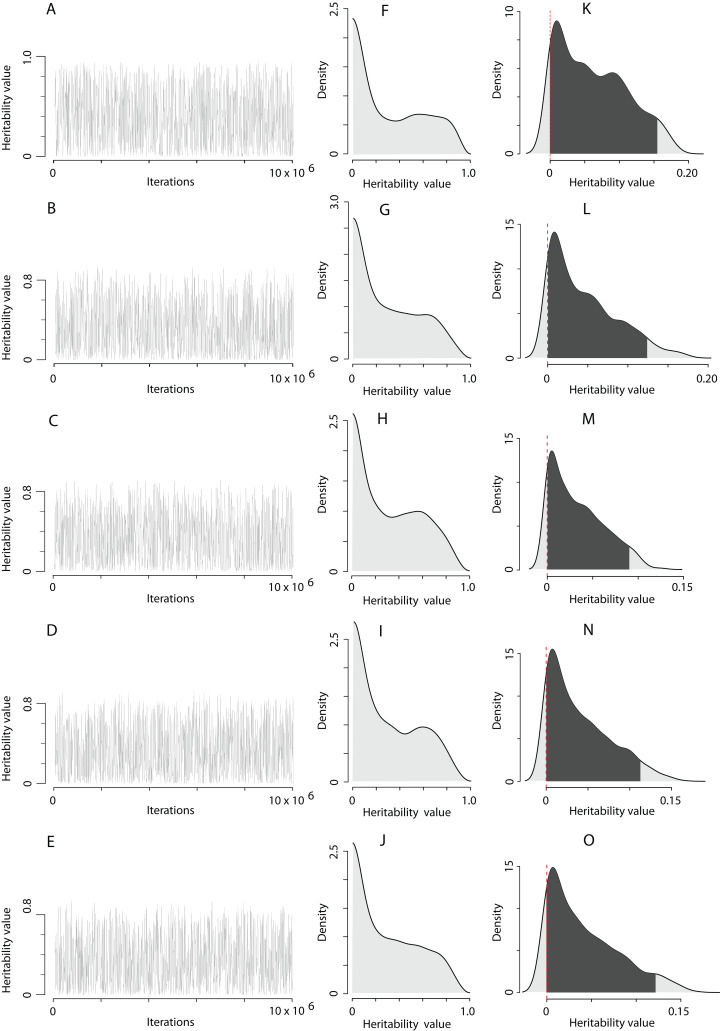
The posterior distribution of the heritability of the EPO number approximated by the MCMC algorithm using the prior R1 for the residual covariance structure. (A–E) Traces of heritability throughout iterations. (F–J) Densities of posterior distributions of heritabilities on a latent scale. (K–O) Densities of posterior distributions of heritabilities on a trait (data) scale. Each line of the figure, consisting of three figure parts, corresponds to the model number in Table 1 (e.g. line 2, containing parts (B), (G), and (L), corresponds to the model 2).

The computation of the trait scale heritability using the QGglmm package after the MCMC algorithm with the prior R1 is shown in Table 1, and the posterior distribution of heritability on the trait and the latent scales is illustrated in Fig. 2. The MCMC algorithm was converged. However, the range of heritability estimates on the latent scale in fact covered the entire range of possible values for this parameter, although most of the estimates have a value close to zero (Figs. 2F–2J). All models detect the presence of the additive genetic variance (Table 1), and a very high residual variance actually equates heritability to zero (the residual variance can be easily calculated as ${\rm{\sigma}}_A^2/{h^2} - {\rm{\sigma}}_A^2$ according to the data in Table 1 for each model). It can also be clearly seen that adding the territory quality to the model as random effect reduces the assessment of heritability by 1.5‒3 times (compare, heritability in model 1 and models 2–5 in Table 1). It seems that the Poisson integer distribution, which the EPO number represents, does not provide enough information for the MCMC algorithm to infer liability variance. The EPO number is an integer categorical variable fitting overdispersed Poisson distribution. In models with categorical response variables there often might not be enough data to fit the variance components of a residual covariance matrix well. For example, in our case, individuals with EPO equal to 5 or 6 may not be presented in all combinations of explanatory variables. Also, in the case of categorical response variables, the fitting process is likely to be not very good, which usually means more computation time and less reliability. In our case, for example, we had to use colossal computing just to keep the autocorrelations at an acceptable level. Without fixing the residual variance, the MCMC algorithm tries to estimate it and faces the lack of some values. In the case of fixation of the residual variance, the MCMC algorithm must no longer explore the residual variance parameter. Therefore, we fixed the residual variance to 1 in the prior R2, though this is usually recommended for binary variables (Reid et al., 2011b; De Villemereuil, Gimenez & Doligez, 2013). Although, in itself, it does not seem to pose any serious problems, however, we just have to be careful about how we present the results. Results in Table 2 could not be correctly interpreted without putting them in the context of the assumed residual variance (Hadfield, 2018). We used fixation of the residual variance as a method to uncover and explore the limits of variation of additive variance. For this purpose, it is more appropriate to fix the residual variance at zero, but MCMCglmm will not mix under this condition (a variance component cannot overlap zero). It seems that mixing properties improve as residual variance increases, however at some point problems with underflow/overflow begin to arise (Van Dyk & Meng, 2001). Thereby, it is a compromise solution to fix the residual variance at 1 using MCMCglmm. Hence, fixing the residual variance at 1, we follow the recommended practice of using the MCMCglmm algorithm (De Villemereuil, 2012; Hadfield, 2018) and make our results more comparable with previously obtained similar results (Reid et al., 2011b).

**Table 1:** Trait scale narrow-sense heritability, *h*^2^, and additive genetic variance, σ^2^_A_, in the EPO number and the simulated null trait computed using the prior R1 for the residual covariance structure.

Trait	Model	Effects		DIC	*h*^2^ (95% CI)	${\rm{\sigma}}_A^2$ (95% CI)
		Fixed	Random			
EPO number	1	~1	~animal	617.2	0.020 [≈0–0.157]	0.038 [≈0–0.93]
	2	~1	~animal + idh(Sex):Nest.Q.Nrec	615.9	0.013 [≈0–0.124]	0.064 [≈0–2.09]
	3	~Age’	~animal + idh(Sex):Nest.Q.Nrec	613.6	0.009 [≈0–0.121]	0.051 [≈0–1.73]
	4	~Age + Age:Sex	~animal + idh(Sex):Nest.Q.Nrec	613.3	0.010 [≈0–0.113]	0.044 [≈0–1.86]
	5	~Age + Sex + Age:Sex	~animal + idh(Sex):Nest.Q.Nrec	613.3	0.006 [≈0–0.122]	0.036 [≈0–1.82]
Simulated EPO number	1	~1	~animal	584.2	0.017 [≈0–0.165]	0.006 [≈0–0.10]
	2	~1	~animal + idh(Sex):Nest.Q.Nrec	585.2	0.014 [≈0–0.147]	0.007 [≈0–0.15]
	3	~Age	~animal + idh(Sex):Nest.Q.Nrec	586.2	0.015 [≈0–0.158]	0.007 [≈0–0.15]
	4	~Age + Age:Sex	~animal + idh(Sex):Nest.Q.Nrec	587.1	0.019 [≈0–0.149]	0.009 [≈0–0.16]
	5	~Age + Sex + Age:Sex	~animal + idh(Sex):Nest.Q.Nrec	587.1	0.040 [≈0–0.145]	0.021 [≈0–0.14]

**Note:**

Fixed and random effects formulas written in R formulation; DIC is the deviance information criterion; 95% CI is the credible intervals calculated as highest posterior density regions; means that fixed effect has pMCMC value ≈ 0.05; Nest.Q.Nrec is quality of breeding site estimated as number of recruits.

**Table 2:** Trait scale narrow-sense heritability, *h*^2^, and additive genetic variance, *σ*^2^_A_, in the EPO number and the simulated null trait computed using the prior R2 for the residual covariance structure.

Trait	Model	Effects		DIC	*h*^2^ (95% CI)	${\rm{\sigma}}_A^2$ (95% CI)
		Fixed	Random			
EPO number	1	~1	~animal	619.7	0.109 [0.067–0.110]	0.216 [0.017–1.20]
	2	~1	~animal + idh(Sex):Nest.Q.Nrec	618.4	0.080 [0.026–0.110]	0.302 [0.015–2.67]
	3	~Age’	~animal + idh(Sex):Nest.Q.Nrec	616.0	0.082 [0.027–0.104]	0.263 [0.028–2.24]
	4	~Age + Age:Sex	~animal + idh(Sex):Nest.Q.Nrec	615.9	0.065 [0.022–0.101]	0.279 [0.007–2.68]
	5	~Age + Sex + Age:Sex	~animal + idh(Sex):Nest.Q.Nrec	616.0	0.065 [0.020–0.101]	0.342 [0.009–2.53]
Simulated EPO number	1	~1	~animal	582.1	0.012 [≈0–0.083]	0.004 [≈0–0.06]
	2	~1	~animal + idh(Sex):Nest.Q.Nrec	582.9	0.016 [≈0–0.071]	0.008 [≈0–0.09]
	3	~Age	~animal + idh(Sex):Nest.Q.Nrec	583.9	0.009 [≈0–0.077]	0.005 [≈0–0.09]
	4	~Age + Age:Sex	~animal + idh(Sex):Nest.Q.Nrec	584.8	0.007 [≈0–0.078]	0.004 [≈0–0.09]
	5	~Age + Sex + Age:Sex	~animal + idh(Sex):Nest.Q.Nrec	584.9	0.012 [≈0–0.073]	0.006 [≈0–0.08]

**Note:**

Fixed and random effects formulas written in R formulation; DIC is the deviance information criterion; 95% CI is the credible intervals calculated as highest posterior density regions; means that the fixed effect has pMCMC value ≈ 0.05; Nest.Q.Nrec is quality of breeding site estimated as number of recruits.

**Figure 3:**
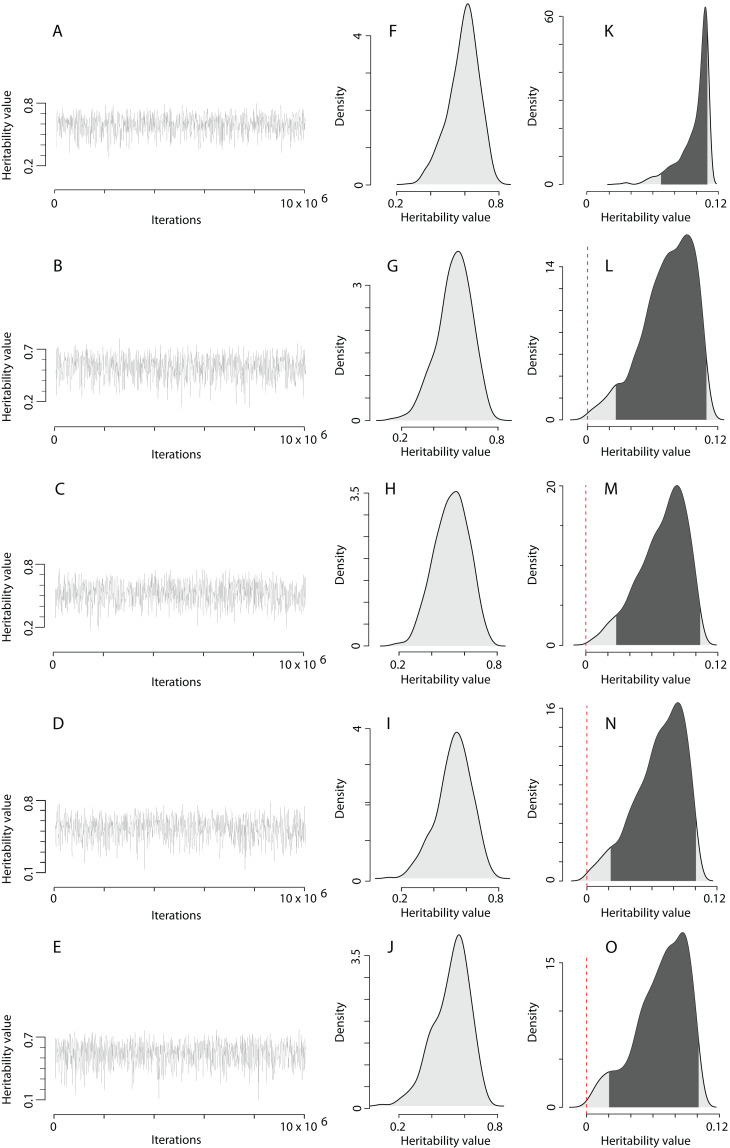
The posterior distribution of the heritability of the EPO number approximated by the MCMC algorithm using the prior R2 for the residual covariance structure. (A–E) Traces of heritability throughout iterations. (F–J) Densities of posterior distributions of heritability on a latent scale. (K–O) Densities of posterior distributions of heritability on a trait (data) scale. Each line of the figure, consisting of three figure parts, corresponds to the model number in Table 2 (e.g. line 3, containing parts (C), (H), and (M), corresponds to the model 3).

**Figure 4:**
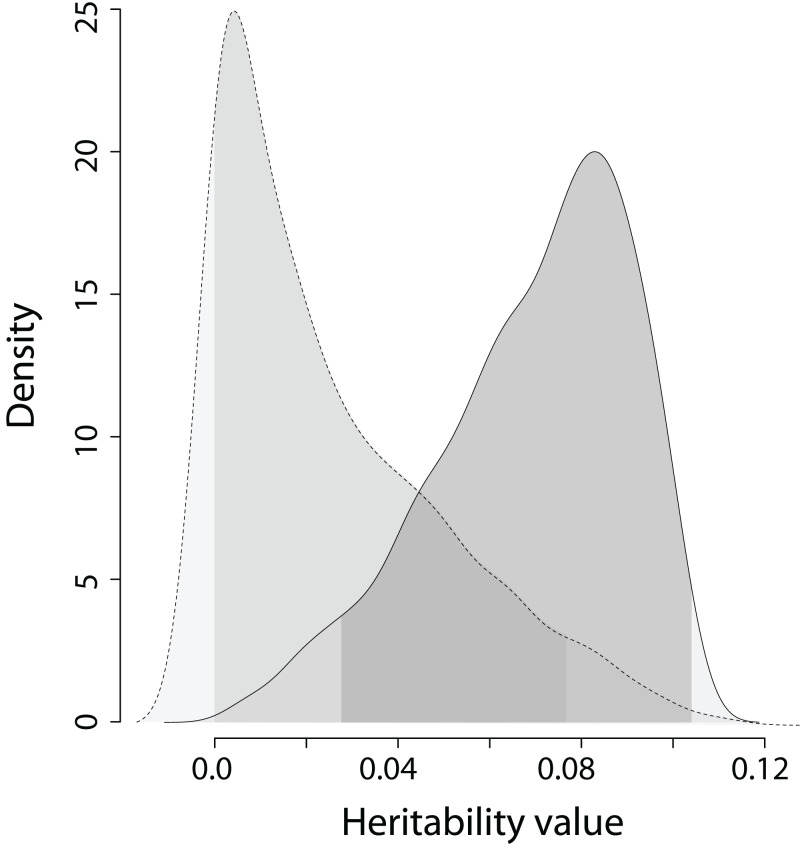
The posterior distribution of the heritability of the EPO number and the heritability of the simulated trait on trait scale for model 3 in Table 2. Dashed line is the density of the heritability of simulated trait; solid line is the dencity of the heritability of the EPO number. The areas under the lines shaded in grey denote 95% credible intervals calculated as highest posterior density regions. Wilcoxon rank sum test statistics (two sided): *V* = 892,510, *p* < 0.0001 (*n* = 1,000).

The heritability estimates on the trait scale using the QGglmm package after the MCMC algorithm with the prior R2 are shown in Table 2, and the posterior distribution of heritability on the trait scale and the latent scale is presented in Fig. 3. The fixation of residual variance increased the estimated values of the additive genetic variance and heritability (Table 2). The upper limit of the 95% credible interval for heritability on the trait scale in all models does not exceed 0.11. An about 1.5 times decrease in heritability estimates is also preserved in models containing the common environment random factor estimated through the number of recruits produced at a breeding site. The mode of the posterior distribution of heritability values of the EPO number is approximately 6.5–10.9% (Table 2). It should be recalled that Table 2 shows the results of MCMCglmm with fixed residual variance, so we considered it as the upper bounds for point estimates (modes in our case) of heritabilities. For the simulated trait, the estimates of the additive genetic variance for all models are low (effectively zero), and the heritability of the null trait is about 1–2% (the 95% CI for a variance component cannot overlap zero, thus, it is impossible to obtain exactly zero heritability and additive variance values). Formal statistical testing showed that the posterior distributions of the heritability of the EPO number, obtained by models listed in Table 2, differ from similar distributions of a simulated trait (Wilcoxon rank sum test (two-sided), *p* < 0.001 for all models; please, see Fig. 4 as an example of the posterior distributions of heritabilities of observed and simulated traits for model 3 from Table 2).

We did not use conventional model selection but constructed a series of animal models differing in their fixed effects structure to investigate fluctuation of the additive genetic variance. Therefore, the downward bias of heritability estimates in univariate models with sex fitting as a fixed factor (Wolak, Roff & Fairbairn, 2015) is not critical, because we also analysed estimates of heritability inferred from simplest models without fixed effects. We are exploring the possible limits of variation in additive genetic variance in general rather than concentrating on finding a single model that we believe should provide a ‘true unbiased’ estimate of heritability. However, it is worse to check the bias in the joint estimates of the additive genetic variances caused by sex fitted as a fixed effect.

The sex-specific estimates of heritabilities and associated additive genetic variances are presented in Tables S1 and S2 in Supplemental Materials. Data shows that there is a downward bias, but it is very small if we consider between-sex differences in heritabilities and the variation in this estimate between models. This bias is clearly visible only if we analysed averaged point estimates of heritabilities. For example, the averaged point estimate (mode) of EPO heritability in females in models without fixation residual variance is about 0.009 and in males—about 0.007 (Table S1), but the univariate model fitted sex as a fixed factor estimated average heritability as 0.006 (Table 1, model 5). The same is true for the models with fixed residual variance. The mode of EPO heritability for females is 0.099 on average, and for males—0.087 on average (Table S2), but the joint estimate of the EPO heritability is 0.065 (Table 2, model 5).

Thus, the point estimate (mode) of the narrow-sense heritability of the EPO number in the pied flycatcher assessed by an animal model is very low: depending on the model used, the point estimate of the heritability (mode) varied from 0.005 to 0.11, and the bounds of the 95% confidence interval are [0–0.16] in the widest range.

## Discussion

The point estimate of heritability depends on the method of its approximation from the posterior distribution. To obtain a point estimate of heritability, there may be used such summary statistics as mean (De Villemereuil, 2012) or mode (Wilson et al., 2010). If the distribution is bell-shaped and symmetric (e.g. as in the case of a normal distribution), then both summary statistics will give similar estimates. However, in the case of more complex distributions (skewed, multimodal, with rare large value outliers) they will be very different. Therefore, we recommend that studies of heritability estimation explicitly report which summary statistics or R package was used so that valid comparison can be performed.

Our study shows that the EPO number has a low heritability in the pied flycatcher nesting in Western Siberia, and with some modelling methods, we could get zero values. It is worth highlighting that, even with the residual variance truncating (fixed to 1) in the models, we cannot get very high heritability values (Table 2). This means that in the observed phenotypic changes of the EPO number, a very small proportion is due to heritable genetic variation, namely additive genetic variance. Nevertheless, according to the definition, near zero or low heritability does not mean that the additive genetic variance is absent (Table 2). Therefore, it does not mean that selection is unable to change the average value of such a trait. However, the response to natural or artificial directional selection on the phenotype is proportional to $h \times {{\rm{\sigma}}_A}$ (Visscher, Hill & Wray, 2008), so both the heritability and genetic variance are important.

This can be illustrated calculating the selection intensity and the selection gradient for a hypothetical shift in the mean trait value. To change the average value of the EPO number by 0.1 ($\Delta \bar z = 0.1$), which equals 2.9% to an increase in the EPO number in the population, the selection intensity $\left(i = \Delta \bar z/\left( {{h^2} \times \sqrt {{\rm{\sigma}}_z^2} } \right)\right)$ should be 1.1–1.6 assuming the heritability equals 0.07–0.1. Such selection intensity values acting constantly in one direction can be maintained only in carefully planned breeding programmes (Preisinger & Flock, 2000). In natural populations, long term constant selection is hardly possible because environments, which determine the directions and magnitudes of selection coefficients, fluctuate rather unpredictably (Grant & Grant, 2002). The selection gradient must be equal to ≈0.36 to facilitate such a change in the EPO number. This value is 2.97 times greater than the modal value of the selection gradient in natural populations of vertebrates (mainly birds and lizards) (Kingsolver & Diamond, 2011). It is several times larger than the modal value of the selection gradient acting in natural populations for such components of fitness as a mating success, survival and fecundity. It is only comparable with the selection gradient revealed for the total fitness of individuals in natural populations (Kingsolver et al., 2001; Kingsolver & Diamond, 2011). The above calculations use the largest point estimates of heritability (Table 2; Table S2), and if we take the point estimates of the heritability obtained without fixing the residual variance (Table 1; Table S1), the natural selection force required to change the trait by 0.1 ($\Delta \bar z = 0.1$) will be most unrealistic. Therefore, we think that the ability of selection acting on the EPO number to have a significant impact on the evolution of extra-pair mating behaviour raises very serious doubts in the case of the pied flycatcher.

Since not many estimates of the heritability of EPCs in socially monogamous species are reported, it seems premature to conduct a sophisticated comparative analysis. However, both studies of the genetic variances of EPCs in *F. hypoleuca* and *M. melodia* show low heritability. It is still early to judge by how low EPC heritability is characteristic of socially monogamous species, but these first estimates make such an assumption very likely (see “Conclusion”). It should additionally be noted that 95% credible intervals of *h*^2^ obtained in our work are narrower (they overlay only 10–16% of the admitted region of *h*^2^) than in the previous studies, where they cover 20–30% of the admitted region of *h*^2^ (Reid et al., 2011a, 2011b). Since the sampling variance of *h*^2^ estimate depends on the number of families (Falconer & MacKay, 1996), it is very likely that narrower 95% credible intervals are due to the substantial number of families (although the families themselves are minor) in the pedigree we used. It also could be the consequence of fact that purely additive genetic effects are not critically dependent on pedigree structures which almost all could provide the same information about the heritability of a quantitative trait (Ekstrøm, 2009).

Humans have a broad-sense heritability of EPCs (the proportion of variation accounted for both additive and nonadditive genetic factors) which in average is 0.53 for both sexes (Zietsch et al., 2015). The authors also estimated the proportion of the additive genetic variance in both sexes to be equal to zero (Zietsch et al., 2015). However, they pointed out that the relative proportions of additive and nonadditive genetic variances should be interpreted cautiously, because authors had little power to distinguish additive and nonadditive genetic effects in this study (Zietsch et al., 2015). It is very likely that human narrow-sense heritability of EPCs is as close to zero as that of birds (see “Conclusion”). However, it cannot be ruled out that human narrow-sense EPCs heritability can be much higher than that of birds due to exceptionally low residual variance (Zietsch et al., 2015), which is typically caused by the fluctuations of environmental factors. For the modern European society, apparently, a strong variability of living conditions of people (both economic and social), as a whole, is not characteristic, and the living conditions themselves, if not close to the optimum, are very standardised.

On the bright side, traits may have low heritability because of increased residual variance due to the multiple non-genetic factors influencing their expression, rather than their association with fitness (Wheelwright, Keller & Postma, 2014). The main sources of variance of the EPO number in the pied flycatchers breeding in Western Siberia are the age of individuals, the quality of the nesting site, and other unknown factors increasing the residual variance. In general, the proportion of birds with EPO among individuals older than 1 year is 49% higher than the same proportion among 1-year-old birds. The proportion of females with EPO among individuals nesting in high quality areas is 68.9% higher than the proportion of such individuals among females nesting in low quality areas in which the number of recruits is 0–2 estimated for the 9-year interval. At present, it seems that extra-pair mating behaviour among pied flycatchers is a plastic phenotypic mating tactic or a low-heritable flexible behaviour rather than the alternative breeding strategy with genetic differentiation among individuals (Gross, 1996) whose expression and consequences depend on the environment and specimen ontogenesis. In a previous study, we have shown that multiple mating (polygyny and EPCs) in the pied flycatcher can be strongly modified depending on the overall properties of the specific environment (Grinkov et al., 2018). For example, in the city where spatial heterogeneity is higher, a lower bird breeding density exists, contact between individuals is difficult and there is probably a lack of males, the incidence of polygyny is 8.5 (!) times higher and the occurrence of EPP is 3.3 times lower for females and 1.7 times lower for males compared to forest habitats (Grinkov et al., 2018). This broad phenotypic plasticity is observed between individuals of two study sites, spaced only 13 km apart, within the same population of the species (Grinkov et al., 2018). On the other hand, the above mentioned attributes of the EPO number (its high plasticity and its responsiveness to environmental changes) may indicate that individuals of the pied flycatcher have no ‘inclination’, ‘propensity’ or ‘craving’ for the EPC in either males or females (Grinkov et al., 2018). EPC as a phenomenon according to its definition is represented in the pied flycatcher population, but there may be no EPC in individuals as an alternative breeding strategy to mate with an extra-pair partner.

Currently, there has been growing recognition that the evolution of diverse mating systems (monogamy and extra-pair copulations, polygamy, promiscuity and others) in more general meaning is governed by the same mechanisms and factors. As an example, it is a classic view that mating systems can be caused by the abilities of individuals to monopolise resources and mates: monogamy is seen as the consequence of the inability of members of either sex to monopolise more than a single mate (Emlen & Oring, 1977; Oring, 1982; Beletsky et al., 1995). One of the significant arguments among others in support of this view and similar (that evolution of distinct mating systems would not be fundamentally different) is the difficulty in classifying mating systems in birds (Grinkov et al., 2018). For example, very often social monogamy occurs as serial monogamy. Serial monogamy implies that social bonds in a couple are formed consecutively for only one reproductive attempt or breeding season (Carey & Nolan, 1975). Thus, the genetic consequences of serial monogamy for specimen living for several years differ little from polygamy: one male, for example, fertilises the eggs of several females (Ford, 1983). Besides, species with serial monogamy can have facultative polygamy (and the pied flycatcher among them). In its most general meaning, the facultative polygamy of serially monogamous species can be viewed as the realisation of serial monogamy within the one reproductive season (Ford, 1983; Grinkov et al., 2018). In socially monogamous species of birds, extra-pair copulations and facultative polygamy are sometimes regarded as one of the types of ‘mate infidelity’ (Ford, 1983). However, it is often very difficult to decide which system is the main scheme of interrelationships between individuals (Ford, 1983), since some bird species comprise both purely monogamous and predominantly polygynous populations (De Santo et al., 2003). Therefore, it seems quite natural today that the hypotheses explaining the EPC evolution (Lifjeld et al., 2019; Brouwer & Griffith, 2019) largely overlap with those explaining the polygamy evolution (Parker & Birkhead, 2013), since ultimately both EPC and polygamy are multiple mating.

In this context, studies that assess the genetic variances of traits associated with female multiple mating are remarkably interesting. In contrast to the estimates of the EPC heritability in socially monogamous species, the estimates of heritability of female multiple mating from species that are not socially monogamous were done much more frequent (Torres-Vila et al., 2002; Linder & Rice, 2005; Evans & Simmons, 2008; McFarlane et al., 2011; Evans & Gasparini, 2013; Travers, Simmons & Garcia-Gonzalez, 2016). Also, there is valuable data on the components of genetic variances of female mating frequency and sperm competitiveness, obtained in the studies of the socially non-monogamous species. The point is that one of the explanations of the evolution of polygamy, which received much attention, focused on the acquisition of sexual or good genes for offspring (Yasui, 1997, 1998). This explanation is also very common in the studies of the socially monogamous species (Brouwer & Griffith, 2019). Analogous to the good genes and sexy sons (Weatherhead & Robertson, 1979; Walker, 1980; Yasui, 1997), the good sperm and sexy sperm hypothesis posits that females can accrue genetic quality for their offspring if males of a superb genetic quality win in sperm competition because the offspring sired by these successful males will exhibit high viability or high sperm competitiveness (Yasui, 1997, 1998). However, selection for polyandry via the sexy sperm and good sperm mechanism is possible only when there is additive genetic variance in both female mating frequency and sperm competitiveness and when these traits exhibit genetic covariance (Evans & Simmons, 2008). Using the *Drosophila melanogaster* model system, it has been shown that female mating frequency can respond to selection due to high heritability (>0.5); however, there has been found low and nonsignificant additive genetic variance in sperm competitiveness and no genetic covariation between sperm competitiveness and polyandry (Travers, Simmons & Garcia-Gonzalez, 2016). It is, therefore, suggested that the sexy sperm and good sperm mechanisms are unlikely to contribute to the evolution of polyandry in this population of *D. melanogaster*.

This example demonstrates the high importance of the key genetic and phenotypic variances and covariances for testing hypotheses of polygamy evolution. However, if we agree that the evolution of diverse mating systems is not fundamentally different, there is a good rationale to take into account the findings of the studies of the socially non-monogamous species in the studies of the socially monogamous species.

## Conclusions

We believe that knowledge of the EPO number heritability will allow a rigorous estimation of balance of all components of direct and indirect selection acting on males and females of the pied flycatcher. This will enable rigorous testing of many specific hypotheses about the origination and maintenance of the EPC and will contribute to a comprehensive understanding of the evolution of extra-pair mating behaviour in socially monogamous species.

The accuracy of a heritability estimate depends on the sample size and pedigree structure. The sampling variance of the estimate of heritability is inversely proportional to the relationship of individuals squared, the number of families and some other factors (Falconer & MacKay, 1996). Hundreds of observations are needed to obtain a standard error less than 0.1, and thousands are needed to attain more precise estimates (Visscher, Hill & Wray, 2008). In our work, the heritability of the EPO number was evaluated for only one reproductive cycle. Therefore, to obtain more precise estimates of the heritability of the extra-pair mating behaviour in the pied flycatcher, multi-generation longitudinal studies are necessary (Reid et al., 2011a, 2011b).

Because the heritability is the proportion of phenotypic variance that is due to genetic factors, it is a population parameter and depends on population-specific factors, such as segregation in a population of the alleles that influence the trait, allele frequencies, the effects of gene variants, and variation due to environmental factors. Therefore, the heritability in one population does not, in theory, predict the heritability of the same trait in another population or other species (Visscher, Hill & Wray, 2008). Nevertheless, in practice, heritabilities of similar traits are often remarkably similar among other populations of the same species, or even across species (Visscher, Hill & Wray, 2008). Therefore, future works, in which the heritability of the EPO number in other populations of the pied flycatcher will be assessed, are thought to be very interesting, although the probability of obtaining completely different values of the heritability of this trait seems to be quite low.

## Supplemental Information

10.7717/peerj.9571/supp-1 Supplemental Information 1 Trait scale narrow-sense heritability, *h^2^*, and additive genetic variance, σ^2^_A_, in the EPO number computed separately for males and females using the prior R1 for the residual covariance structure. Fixed and random effects formulas written in R formulation; DIC is the deviance information criterion; 95% CI is the credible intervals calculated as highest posterior density regions; * means that fixed effect has pMCMC value < 0.05; Nest.Q.Nrec is quality of breeding site estimated as number of recruits.

10.7717/peerj.9571/supp-2 Supplemental Information 2 Trait scale narrow-sense heritability, *h^2^*, and additive genetic variance, σ^2^_A_, in the EPO number computed separately for males and females using the prior R2 for the residual covariance structure. Fixed and random effects formulas written in R formulation; DIC is the deviance information criterion; 95% CI is the credible intervals calculated as highest posterior density regions; * means that fixed effect has pMCMC value < 0.05; Nest.Q.Nrec is quality of breeding site estimated as number of recruits.

10.7717/peerj.9571/supp-3 Supplemental Information 3 Raw data. The number of extra-pair offspring and the values of all fixed and random factors used in the calculations and the pruned (informative) pedigree.

## Additional Information and Declarations

### Data Availability

The following information was supplied regarding data availability: Raw data is available in the Supplemental File.
