# Supplementary material for: Heritability of the extra-pair mating behaviour of the pied flycatcher in Western Siberia
Source: PeerJ. 2020 Jul 31;8:e9571. doi: 10.7717/peerj.9571 (PMC7397985; doi:10.7717/peerj.9571)
Supplement: Supplemental Information 1 — Fixed and random effects formulas written in R formulation; DIC is the deviance information criterion; 95% CI is the credible intervals calculated as highest posterior density regions; * means that fixed effect has pMCMC value < 0.05; Nest.Q.Nrec is quality of breeding site estimated as number of recruits. [file peerj-08-9571-s001.docx]

**Table S1**. Trait scale narrow-sense heritability, $h^{2}$, and additive genetic variance, $\sigma_{A}^{2}$, in the EPO number computed separately for males and females using the prior R1 for the residual covariance structure.

| Sex | Model No | Effects | | DIC | $h^{2}$ (95% CI) | $\sigma_{A}^{2}$ (95% CI) |
| --- | --- | --- | --- | --- | --- | --- |
|  |  | Fixed | Random |  |  |  |
| Female | 1 | ~1 | ~animal | 348.3 | 0.010 (0.0 - 0.163) | 0.719 (0.0 - 3.05) |
|  | 2 | ~1 | ~animal + Nest.Q.Nrec | 347.8 | 0.008 (0.0 - 0.153) | 1.141 (0.0 - 3.52) |
|  | 3 | ~Age | ~animal | 347.4 | 0.010 (0.0 - 0.155) | 0.894 (0.0 - 3.67) |
|  | 4 | ~Age | ~animal + Nest.Q.Nrec | 346.9 | 0.009 (0.0 - 0.139) | 1.872 (0.0 - 5.32) |
| Male | 1 | ~1 | ~animal | 269.3 | 0.008 (0.0 - 0.142) | 0.216 (0.0 - 1.02) |
|  | 2 | ~1 | ~animal + Nest.Q.Nrec | 268.4 | 0.008 (0.0 - 0.133) | 2.002 (0.0 - 3.04) |
|  | 3 | ~Age^*^ | ~animal | 267.8 | 0.007 (0.0 - 0.135) | 0.221 (0.0 - 1.32) |
|  | 4 | ~Age^*^ | ~animal + Nest.Q.Nrec | 266.7 | 0.005 (0.0 - 0.113) | 1.053 (0.0 - 2.34) |

Fixed and random effects formulas written in R formulation; DIC is the deviance information criterion; 95% CI is the credible intervals calculated as highest posterior density regions; * means that fixed effect has pMCMC value < 0.05; Nest.Q.Nrec is quality of breeding site estimated as number of recruits.
