# Supplementary material for: Heritability of the extra-pair mating behaviour of the pied flycatcher in Western Siberia
Source: PeerJ. 2020 Jul 31;8:e9571. doi: 10.7717/peerj.9571 (PMC7397985; doi:10.7717/peerj.9571)
Supplement: Supplemental Information 2 — Fixed and random effects formulas written in R formulation; DIC is the deviance information criterion; 95% CI is the credible intervals calculated as highest posterior density regions; * means that fixed effect has pMCMC value < 0.05; Nest.Q.Nrec is quality of breeding site estimated as number of recruits. [file peerj-08-9571-s002.docx]

**Table S2**. Trait scale narrow-sense heritability, $h^{2}$, and additive genetic variance, $\sigma_{A}^{2}$, in the EPO number computed separately for males and females using the prior R2 for the residual covariance structure.

| Sex | Model No | Effects | | DIC | $h^{2}$ (95% CI) | $\sigma_{A}^{2}$ (95% CI) |
| --- | --- | --- | --- | --- | --- | --- |
|  |  | Fixed | Random |  |  |  |
| Female | 1 | ~ 1 | ~ animal | 349.9 | 0.109 (0.038 - 0.112) | 0.292 (0.002 - 3.731) |
|  | 2 | ~ 1 | ~ animal + Nest.Q.Nrec | 349.4 | 0.089 (0.024 - 0.110) | 0.335 (0.002 - 6.908) |
|  | 3 | ~ Age | ~ animal | 348.9 | 0.104 (0.039 - 0.107) | 0.301 (0.009 - 3.793) |
|  | 4 | ~ Age | ~ animal + Nest.Q.Nrec | 348.4 | 0.094 (0.025 - 0.106) | 0.365 (0.001 - 6.075) |
| Male | 1 | ~ 1 | ~ animal | 272.2 | 0.103 (0.019 - 0.106) | 0.091 (0.000 - 1.722) |
|  | 2 | ~ 1 | ~ animal + Nest.Q.Nrec | 271.1 | 0.070 (0.009 - 0.105) | 0.128 (7.271 - 3.644) |
|  | 3 | ~ Age^*^ | ~ animal | 270.6 | 0.093 (0.017 - 0.096) | 0.088 (0.000 - 1.535) |
|  | 4 | ~ Age^*^ | ~ animal + Nest.Q.Nrec | 269.6 | 0.081 (0.009 - 0.096) | 0.122 (5.388 - 2.731) |

Fixed and random effects formulas written in R formulation; DIC is the deviance information criterion; 95% CI is the credible intervals calculated as highest posterior density regions; * means that fixed effect has pMCMC value < 0.05; Nest.Q.Nrec is quality of breeding site estimated as number of recruits.
